# Smartphone App–Based Exercise for Pregnant Women in Indonesia: Quasi-Experimental Study on Physical Activity, Fear of Childbirth, and Quality of Life

**DOI:** 10.2196/73105

**Published:** 2025-12-24

**Authors:** Dewi Marfuah, Tukimin Bin Sansuwito, Rathimalar Ayakannu

**Affiliations:** 1 Sekolah Tinggi Ilmu Keperawatan Persatuan Perawat Nasional Indonesia (PPNI) Jawa Barat Bandung, West Java Indonesia; 2 Faculty of Nursing and Allied Health Lincoln University College Petaling Jaya, Selangor Malaysia

**Keywords:** fear of childbirth, mobile health, physical activity, pregnancy, quality of life, smartphone app

## Abstract

**Background:**

Pregnancy, a vital phase in a woman’s life, entails immense physical, psychological, and emotional alterations that might affect maternal health. Physical activity during pregnancy improves health outcomes; however, adherence to the recommendation is low. Moreover, fear of childbirth (FoC) has a negative impact on maternal mental health and quality of life (QoL). Mobile health (mHealth) interventions, especially those delivered through smartphone-based exercise management apps, provide a scalable solution to improve maternal health outcomes.

**Objective:**

This study aims to assess the impact of an exercise management intervention based on a smartphone app on physical activity, FoC, and QoL in pregnant women in Indonesia.

**Methods:**

We used a quasi-experimental design with repeated measures, conducted at public health centers in West Java, Indonesia. A total of 240 pregnant women were recruited through convenience sampling and allocated to either an intervention group (n=120), which received a smartphone app–based exercise and behavioral program, or a control group (n=120), which received standard prenatal care. Data were collected at 3 time points: baseline measurement (T0), postintervention measurement (T1), and 1-month follow-up measurement (T2). The intervention targeted improvements in physical activity, reduction of FoC, and enhancement of QoL. Validated instruments were used to assess outcomes, including the Pregnancy Physical Activity Questionnaire (PPAQ), the Wijma Delivery Expectation Questionnaire Version A (WDEQ-A), and the Quality-of-Life Gravidarum (QOL-GRAV) scale. Statistical analyses were performed using repeated measures ANOVA with Bonferroni post hoc tests, and effect sizes were calculated using Cohen *d*.

**Results:**

The intervention group had significant increases in physical activity levels from T0 to T1 (Cohen *d*=0.65; *P*<.001) and from T0 to T2 (Cohen *d*=0.72, *P*<.001), whereas there were no significant changes in the control group. FoC scores were significantly lower at T1 (Cohen *d*=0.52; *P*<.001) and T2 (Cohen *d*=0.56; *P*<.001) compared to T0 in the intervention group, but no changes were observed in the control group. QoL scores increased significantly in the intervention group from T0 to T1 (Cohen *d*=0.60; *P*<.001) and from T0 to T2 (Cohen *d*=0.68; *P*<.001), while there were no significant changes noted in the control group.

**Conclusions:**

The exercise management intervention using the smartphone app was effective in increasing physical activity, reducing FoC, and improving QoL among pregnant women in Indonesia. The intervention represents a scalable and accessible mechanism through which maternal health can be improved in limited-resource contexts. Large-scale, long-term studies are needed to evaluate the sustainability of the benefits observed and the incorporation of mHealth solutions in standard prenatal management.

## Introduction

### Background

Pregnancy is a transformative period in a woman’s life, marked by substantial physiological, psychological, and emotional changes that can impact maternal well-being and quality of life (QoL) [1]. While these changes are part of normal gestation, they can be accompanied by discomfort, anxiety, and reduced physical activity, particularly during the antenatal period [2]. In many low- and middle-income countries (LMICs), including Indonesia, these challenges are further compounded by limited access to antenatal education, support systems, and personalized care strategies [3]. One critical but often overlooked aspect is the fear of childbirth (FoC), which affects a substantial proportion of pregnant women and is associated with increased stress, lower physical activity levels, and diminished QoL [4,5].

Evidence suggests that promoting maternal physical activity during pregnancy not only improves physical health outcomes but also reduces FoC and enhances emotional well-being [6]. However, in resource-limited settings, structured support for safe and effective antenatal exercise is often lacking. This highlights the need for innovative, culturally appropriate, and scalable interventions, such as mobile app–based programs to support pregnant women in improving their physical and psychological health during pregnancy [7,8].

### Literature Review

Exercise during pregnancy is an established determinant of both maternal and fetal health. Numerous studies have shown that regular physical activity reduces the risk of gestational diabetes, hypertension, and excessive weight gain while also improving mental well-being and sleep quality [9]. Despite these known benefits, physical inactivity during pregnancy remains prevalent. Globally, only 20%-30% of pregnant women meet the recommended guidelines for physical activity, while 70%-80% remain insufficiently active [10]. In Indonesia, the situation is more pronounced, with an estimated 85% of pregnant women failing to meet physical activity guidelines, primarily due to cultural taboos, low health literacy, and limited access to safe exercise options [11].

Alongside low physical activity levels, many pregnant women also experience FoC, a form of anxiety characterized by negative expectations and worry about the birthing process [12]. Globally, FoC affects 20%-25% of pregnant women, with even higher prevalence in LMICs, reaching 30%-40% [13-15]. In Indonesia, FoC is reported in approximately 28%-35% of pregnant women, especially among primiparous mothers and those with prior traumatic deliveries [16]. Contributing factors include inadequate childbirth education, negative cultural perceptions of labor, and limited psychosocial support [17,18]. These issues are exacerbated by underresourced health systems, where access to prenatal counseling and mental health services is often limited [19,20].

FoC not only undermines maternal mental health but is also linked to negative clinical outcomes, including prolonged labor, elective cesarean delivery without medical indication, and increased risk of postpartum depression [21,22]. Consequently, addressing FoC is crucial to improving maternal and neonatal health outcomes, particularly in settings where health care infrastructure and antenatal education remain insufficient.

Physical inactivity and FoC significantly affect a third key dimension: QoL during pregnancy. QoL encompasses physical, emotional, and social well-being and is influenced by factors such as physical health status, psychological resilience, access to health care, and perceived social support [23,24]. Studies in Indonesia have reported that between 45%-65% of pregnant women experience poor QoL, particularly in low-income and rural areas [25]. Factors such as lack of autonomy in self-care, economic hardship, and pressure to prioritize family over personal well-being further compromise their QoL [26,27].

Given these interlinked challenges—low physical activity, high FoC, and poor QoL—integrated and culturally sensitive interventions are urgently needed. Conventional interventions such as prenatal education classes, counseling, and supervised group exercise programs have been implemented to address these issues [28-31]. However, participation remains low due to cultural barriers, logistical constraints, and social stigmas associated with public exercise during pregnancy [9,32]. Many programs are short-lived, rely on external facilitators, and fail to address spiritual or cultural values that shape women’s health behavior [33].

Recent years have witnessed the rise of mobile health (mHealth) technologies, particularly smartphone apps, as scalable solutions for delivering health interventions in LMICs [34,35]. mHealth platforms have been used to promote physical activity, provide mental health support, and deliver educational content for self-care during pregnancy [36]. Several apps, such as MobileMoms, Yourtime, and Smart Fitness, offer prenatal fitness content and personalized workout plans [37-39]. While some include components that address mental well-being and FoC, they often lack sufficient interactivity, real-time feedback, and cultural adaptation [38,40].

Moreover, these existing apps are rarely tailored for LMIC contexts. Many do not offer modesty-sensitive workout options, language localization (eg, Bahasa Indonesia), or safety features for high-risk pregnancies [40,41]. The cost of premium features also excludes low-income users from accessing vital content. Critically, few of these apps have been rigorously evaluated in clinical trials, especially in Indonesia, where no culturally adapted, evidence-based mHealth solutions have yet been validated [41,42]. Therefore, there is a clear gap in digital health interventions that simultaneously promote safe physical activity, reduce FoC, and enhance QoL for pregnant women in resource-constrained settings. A culturally adapted, smartphone-based exercise management program that includes education, motivation, and real-time support may represent a viable, low-cost, and scalable solution to address these interrelated issues in Indonesia and similar LMICs.

To fill this gap, this study developed and evaluated a culturally adapted, smartphone-based intervention that combines structured physical activity modules with motivational interviewing (MI), a behavioral strategy designed to enhance motivation, reduce anxiety, and promote autonomous decision-making. Rather than evaluating mere exposure to the app, the aim of this study was to assess the effectiveness of its core components, specifically the integration of interactive aerobic and resistance exercise training, educational content, and MI–based counselling on improving physical activity levels, reducing FoC, and enhancing QoL among pregnant women in Indonesia. MI was chosen as a key behavior change element because of its client-centered approach that supports individuals in exploring and overcoming ambivalence, setting goals, and building self-efficacy [38,39]. Although MI has demonstrated strong outcomes in various health contexts, its integration into mHealth platforms for antenatal care in LMICs remains limited. By embedding MI into the app’s digital workshops and in-app communication, this intervention addresses not only the physical but also the emotional and psychological dimensions of maternal health.

### Goal of This Study

This study aimed to examine the effects of a smartphone app–based exercise management intervention on physical activity, FoC, and QoL among pregnant women in Indonesia. With smartphones becoming a part of everyday life and mHealth tools gaining popularity, this study set out to explore how a digital intervention could offer a practical, scalable, and accessible way to support better maternal health outcomes, especially in settings where resources are limited.

## Methods

### Study Design and Setting

A quasi-experimental study with a repeated-measures design was conducted at the public health center (PHC) in West Java, Indonesia. There were 240 pregnant women who took exercise management interventions between May and September 2024. Participants were allocated into two groups: an intervention group (n=120) receiving the app-based program and a control group (n=120) receiving standard prenatal care. Data were collected at 3 time points: baseline measurement (T0), postintervention measurement (T1), and 1-month follow-up measurement (T2).

### Smartphone App–Based Exercise Development

The intervention used a smartphone app compatible with Android and Windows mobile platforms to support exercise management for pregnant women. Before development, a comprehensive review of best practices in mHealth technology was conducted using peer-reviewed journal articles, academic books, YouTube educational content from an obstetrician, and podcasts. The app was developed using a client-server architecture based on HTTP and Web API (application programming interface) protocols, supported by a MySQL database and a backend built with Apache Tomcat and the Spring Framework.

**Figure 1:**
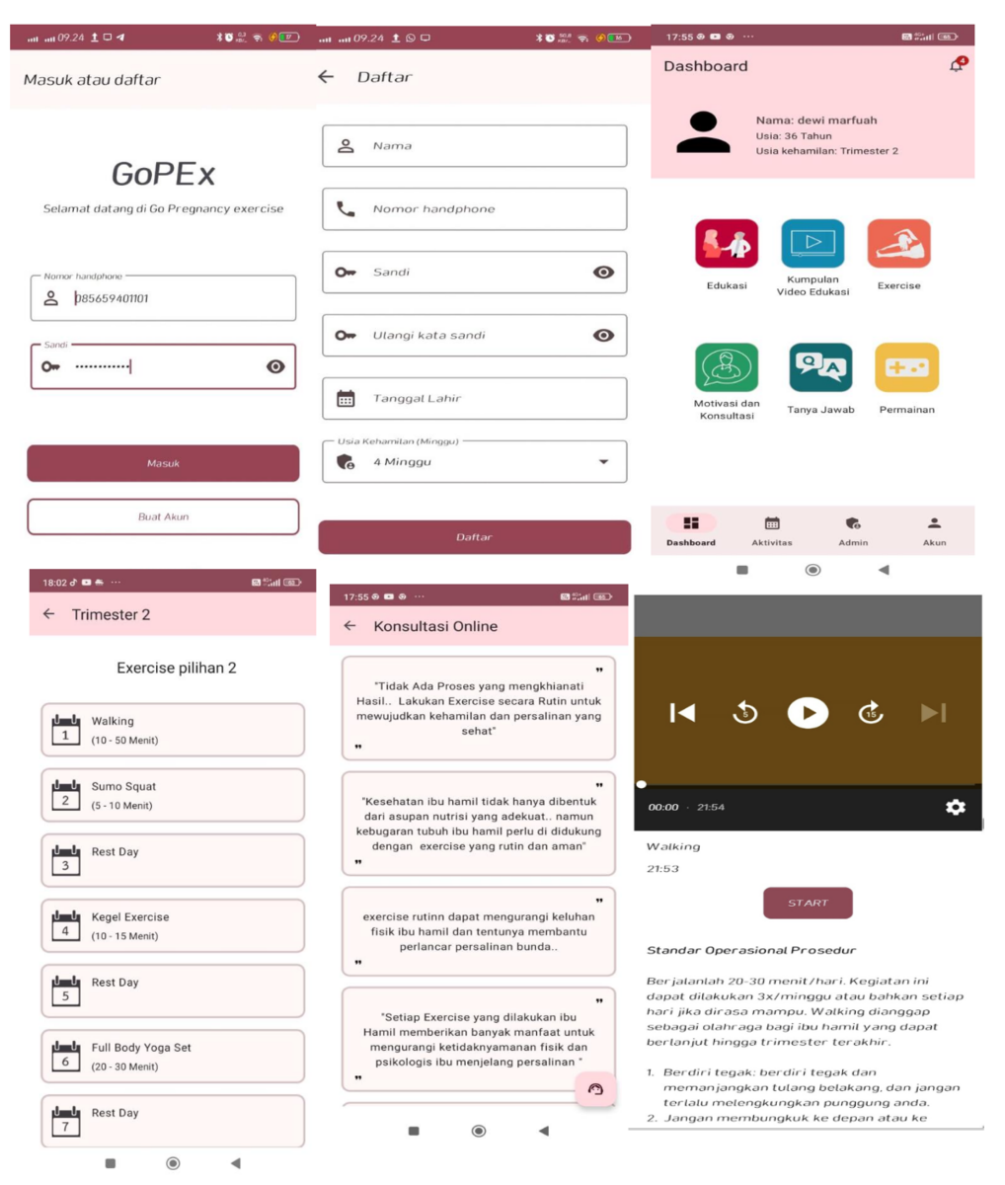
Example of smartphone app-based exercise management.

The mobile application was developed using the Android software development kit (SDK) and featured nine core modules: (1) user ID login, (2) educational content on physical activity tailored to pregnant women accompanied by interactive quizzes, (3) aerobic exercise demonstration video, (4) resistance exercise demonstration video, (5) aerobic exercise training module, (6) resistance exercise training module, (7) motivational interviewing module, (8) a question-and-answer feature, and (9) self-assessment tools for tracking progress (Figure 1).

Beyond delivering exercise content, the app was designed to facilitate behavior change through several integrated mechanisms. Participants used the self-assessment tools to log daily activity duration, intensity, and perceived effort, which supported regular self-monitoring and goal tracking. The MI module included 6 short sessions and personalized in-app messages based on MI techniques, such as reflective listening, affirmations, and open-ended prompts, encouraging participants with messages like “Keep up the great effort—you are doing something meaningful for you and your baby.” These messages aimed to reduce ambivalence and strengthen self-efficacy. Additionally, the app featured brief cognitive and emotional prompts designed to help participants reflect on their progress and identify barriers, drawing conceptually from cognitive-behavioral therapy (CBT) strategies. Together, these interactive components were intended to empower pregnant women to adopt and sustain healthy behaviors throughout the intervention period.

### Intervention Protocol

The intervention was conducted over a 1-month period, during which participants in the intervention group accessed a smartphone app daily. The app featured structured modules, including educational content, aerobic and resistance training videos, interactive self-assessments, and embedded MI strategies. MI was implemented through 6 brief virtual sessions within the app that addressed common psychological barriers to physical activity, promoted self-efficacy, and guided participants in setting achievable exercise goals. In addition, personalized in-app messages delivered by trained facilitators applied MI principles such as reflective listening, affirmations, and open-ended questions to reinforce motivation, explore ambivalence, and support behavioral change.

User engagement was monitored through the app’s backend system, which automatically recorded login frequency, module completion, video views, and digital diary entries. Weekly reminders and individualized follow-ups through WhatsApp and in-app notifications helped maintain adherence. A built-in safety checklist was provided weekly to monitor for symptoms such as musculoskeletal discomfort or fatigue. Reports were reviewed by a nurse or physiotherapist, and follow-up assessments were conducted when needed to ensure participant safety.

### Sample

A total of 240 pregnant women participated in the study, with 120 individuals assigned to the intervention group and 120 to the control group. Inclusion criteria were: pregnant women aged 18 years or older, currently in the first or second trimester, medically cleared and recommended by a health care provider to engage in physical exercise, able to read and write, and willing to provide informed consent and actively participate in the intervention. Exclusion criteria included: diagnosed mental health disorders, pregnancy-related complications such as cardiovascular or pulmonary disease, history of cervical cerclage, multiple pregnancies with elevated risk of preterm labor, placenta previa after 26 weeks of gestation, preterm labor or ruptured membranes, pre-eclampsia or pregnancy-induced hypertension, and severe anemia.

A priori power analysis using G*Power software (version 3.1.9.7; Heinrich-Heine-Universität Düsseldorf) determined that a total sample size of 240 participants (120 per group) was required to detect a medium effect size (Cohen *d*=0.5) with a significance level of α=0.05 and a power of 95% [43]. Participants were recruited through convenience sampling at PHCs in West Java, Indonesia. In practice, this involved collaboration with midwives and antenatal care providers who identified and approached eligible pregnant women during routine antenatal visits. Recruitment was conducted on weekdays during standard clinic hours (08:00-14:00), over a 6-week period. Health care staff introduced the study using a brief script, provided information sheets, and referred interested participants to trained research assistants who conducted eligibility screening and obtained written informed consent. This approach allowed for direct engagement with women already seeking prenatal care, facilitating timely enrollment while accommodating the operational constraints of the clinical setting.

Following recruitment and eligibility screening, participants were sequentially allocated to either the intervention or control group using an alternating assignment approach, ensuring equal distribution. This method was selected due to the practical constraints of the real-world clinical setting, where randomization was not feasible. Group allocation was conducted by research assistants who were not involved in the outcome assessments to minimize allocation bias.

### Instrument

A structured questionnaire designed for this study obtained data on age, education, occupation, gestational age, parity, BMI, living with family, gestational age, history of abortion, employment status, and number of children.

Pregnancy Physical Activity Questionnaire (PPAQ) is a validated 32-item questionnaire used to assess frequency, duration, and intensity of physical activity engaged in during pregnancy [44]. Responses are coded as sedentary, light, moderate, or vigorous activity levels. The English version was found to be highly reliable (Cronbach α=0.82) [44]. To ensure cross-cultural validity, the PPAQ was translated into Bahasa Indonesia in compliance with the cross-cultural adaptation methodology [45], which included 2 independent bilingual translators translating the tool into Bahasa Indonesia. Linguistic and conceptual equivalence was established through the resolution of discrepancies by a committee of obstetricians, public health experts, and linguists. A third translator then retranslated the Indonesian version into English without access to the original version. The Indonesian PPAQ was pilot-tested for clarity and cultural relevance in 30 pregnant women. Structural validity was confirmed by confirmatory factor analysis (CFA; comparative fit index [CFI]=0.92; root-mean-square error of approximation [RMSEA]=0.06). Internal consistency was excellent (Cronbach α=0.79).

Wijma Delivery Expectation Questionnaire Version A (WDEQ-A) is a 33-item Likert-scale (rated 0-5) instrument that measures FoC severity. Total scores can range from 0 to 165. Scoring ranges from 0 to 3 for each item, totaling a composite (5-item) score, with higher scores indicating greater FoC [46].

The Swedish version exhibited high reliability (α=0.87) [46]. The WDEQ-A was translated and adapted with respect to Indonesian culture. Forward-backward translation was conducted independently by translators, followed by the reconciliation of semantic and idiomatic discrepancies. A panel of midwives, psychologists, and obstetricians rated item relevance (Content Validity Index=0.91). About 30 pregnant women completed the translated version and confirmed comprehension through feedback. The Bahasa Indonesia version had good internal consistency (α=0.83) and 2-week test-retest reliability (intraclass correlation coefficient [ICC]=0.85).

Quality-of-Life Gravidarum (QOL-GRAV) is a 25-item questionnaire consisting of 3 domains (physical, emotive, and social) regarding QoL [47]. Scales range from 25 to 100, with higher scores indicating higher QoL. The original version showed good reliability (α=0.89). The QOL-GRAV was adapted according to the framework for cross-cultural validation of Beaton et al [45]. A multidisciplinary team conducted a pilot study to ensure the specificity of the tool was appropriate to the target population and administered to 30 pregnant women, where minor updates were made to improve clarity. CFA confirmed the factor structure (RMSEA=0.07; CFI=0.94). Internal validity of the Indonesian version was strong (α=0.85), and convergent validity measured with Short Form-12 Health Survey showed *r*=0.76.

### Procedure

Participants were recruited in collaboration with PHCs in West Java, Indonesia. During routine antenatal check-ups, health care practitioners screened pregnant women for eligibility based on the inclusion and exclusion criteria. Clinic staff then invited eligible participants to join the study, providing them with an information sheet detailing the study’s objectives, procedures, potential risks and benefits, and the voluntary nature of participation. Baseline data collection was conducted for both the intervention and control groups prior to the start of the intervention. Data were gathered using structured questionnaires administered either electronically or in person by trained research assistants. These assistants were available to offer clarifications to ensure accurate and complete responses. For participants in the intervention group, the program began with a structured educational phase on day 1. This included 6 virtual workshops delivered through the smartphone app, covering safe exercise practices during pregnancy, contraindications, and the fundamentals of MI. Within the app’s interactive platform, participants engaged in moderated small-group discussions (5-10 participants per group) aimed at fostering self-efficacy, goal-setting, and social support.

Participants were instructed to record their daily exercise behaviors, including type, duration, intensity, and perceived exertion using the app’s integrated digital diary feature. The app also delivered automated, personalized motivational messages through an in-app chat function, grounded in MI principles, to reinforce adherence, address barriers, and provide ongoing encouragement from trained facilitators.

The control group received standard antenatal care, which included routine clinical visits and general physical activity advice but did not have access to the smartphone app or any structured behavioral intervention.

Follow-up data collection occurred at one time point: T2, 1 month post baseline, for both groups.

### Data Analysis

Data were analyzed using IBM SPSS Statistics (version 26.0; IBM Corp). Descriptive statistics were used to summarize baseline demographic characteristics, including means and SDs for continuous variables and frequencies and percentages for categorical variables. To evaluate the intervention’s effects over time, repeated-measures analysis of variance (RM-ANOVA) was conducted to assess both within-group and between-group differences in physical activity, FoC, and QoL across 3 time points: baseline (T0), midintervention (T1), and postintervention (T2). Where significant main effects were detected, Bonferroni-adjusted post hoc comparisons were performed to identify specific differences between time points. Effect sizes were calculated using Cohen *d* to quantify the magnitude of the intervention’s impact. To address missing data and preserve the integrity of the randomized design, intention-to-treat analysis was used using multiple imputation techniques. Statistical significance was set at *P*<.05 for all analyses.

### Ethical Considerations

This study received ethical approval from the Institutional Review Board of Sekolah Tinggi Ilmu Keperawatan Persatuan Perawat Nasional Indonesia (STIKep PPNI) Jawa Barat, Indonesia (Approval No III/098/KEPK/STIKep/ PPNI/Jabar/III/2024). All participants provided written informed consent prior to enrollment and were informed of their right to withdraw at any time without consequence. To ensure privacy and confidentiality, all data were anonymized and stored on secure, password-protected servers with regular audits to maintain data integrity. No personal identifiers were linked to the dataset. Participants did not receive monetary or material compensation for participation.

## Results

### Overview

**Figure 2:**
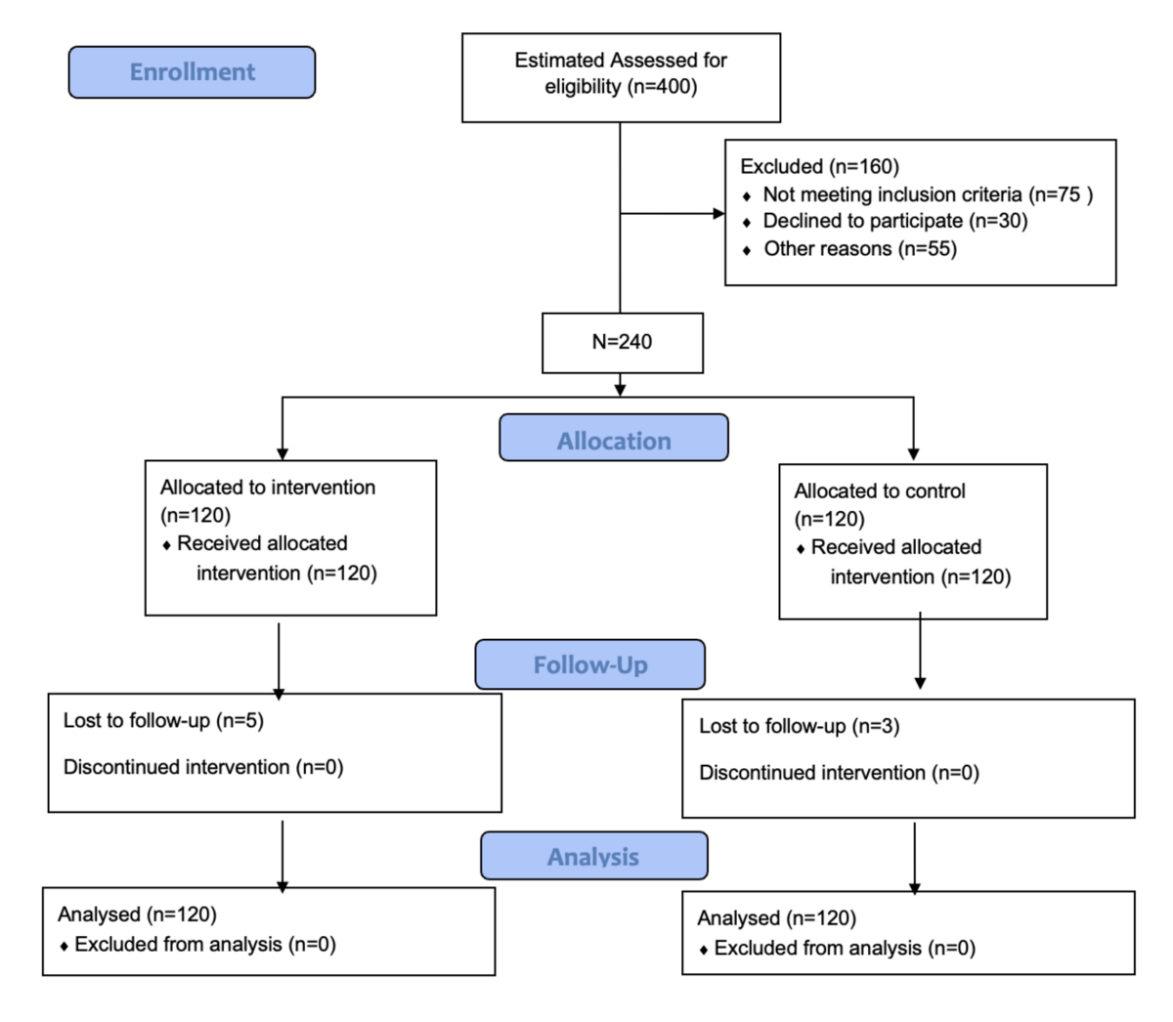
CONSORT (Consolidated Standards of Reporting Trials) 2010 flow diagram.

The CONSORT (Consolidated Standards of Reporting Trials) flow diagram (Figure 2) outlines the progression of participants through the key phases of the study. Among the 400 individuals assessed for eligibility, 160 (40%) were excluded, including 75 (18.8%) who did not meet the inclusion criteria, 30 (7.5%) who declined to participate, and 55 (13.8%) excluded for other reasons. A total of 240 (100%) participants met the eligibility criteria and were enrolled in the study. Of those enrolled, 120 (50%) participants were allocated to the intervention group and 120 (50%) participants to the control group. All participants in both groups received the intervention as assigned. During the follow-up phase, 5 of 120 (4.2%) participants in the intervention group were lost to follow-up, while none discontinued the intervention. In the control group, 3 out of 120 (2.5%) participants were lost to follow-up, with no participants discontinuing. At the analysis stage, a total of 120 participants in the intervention group and 120 participants in the control group were included in the final analysis. No participants were excluded due to protocol deviations or missing data.

### Demographic Characteristics

Table 1 presents the baseline demographic and clinical characteristics of participants in both the intervention and control groups, each comprising 120 pregnant women. The mean age in the intervention group was 28.4 (SD 4.2) years, while the control group had a mean age of 27.9 (SD 3.8) years, with no statistically significant difference between the groups (*P*=.32). In terms of education level, a total of 48 (40%) participants in the intervention group and 52 (43.3%) participants in the control group had completed high school or less, while 72 (60%) and 68 (56.7%) had attained college or university education, respectively. Employment status showed a similar distribution, with 65 (54.2%) participants employed and 55 (45.8%) unemployed in the intervention group, compared with 58 (48.3%) employed and 62 (51.7%) unemployed in the control group. These differences were not statistically significant (*P*=.28). The mean gestational age was 20.1 (SD 6.3) weeks in the intervention group and 19.8 (SD 5.9) weeks in the control group (*P*=.71). A history of abortion was reported by 18 participants (15%) in the intervention group and 15 (12.5%) participants in the control group, with no significant difference between the groups (*P*=.56). The average number of children was 1.2 (SD 0.9) in the intervention group and 1.3 (SD 1.0) in the control group (*P*=.41). Regarding BMI, 10 (8.3%) participants in the intervention group and 8 (6.7%) in the control group were classified as underweight; 85 (70.8%) participants and 88 (73.3%), respectively, had a normal BMI; and 25 (20.8%) and 24 (20%) were categorized as overweight or obese (*P*=.63). Living arrangements were also comparable, with 112 participants (93.3%) in the intervention group and 115 (95.8%) in the control group living with family (*P*=.39). Overall, no statistically significant differences were observed between the two groups at baseline, indicating that the randomization or group assignment process achieved balanced groups for subsequent analysis.

**Table 1:** Baseline demographic and clinical characteristics of participants.

Characteristic		Intervention group (n=120)	Control group (n=120)	*P* value	
Age (years), mean (SD)		28.4 (4.2)	27.9 (3.8)	.32^a^	
**Education level, n (%)**					.45^b^
	High school or less	48 (40)	52 (43.3)		
	College or university	72 (60)	68 (56.7)		
**Employment status, n (%)**					.28^b^
	Employed	65 (54.2)	58 (48.3)		
	Unemployed	55 (45.8)	62 (51.7)		
Gestational age (weeks), mean (SD)		20.1 (6.3)	19.8 (5.9)	.71^a^	
History of abortion, *n* (%)		18 (15)	15 (12.5)	.56^b^	
Number of children, mean (SD)		1.2 (0.9)	1.3 (1.0)	.41^a^	
**BMI (kg/m²), n (%)**					.63^b^
	Underweight	10 (8.3)	8 (6.7)		
	Normal	85 (70.8)	88 (73.3)		
	Overweight or obese	25 (20.8)	24 (20)		
Living with family, *n* (%)		112 (93.3)	115 (95.8)	.39^b^	

^a^Independent *t* test (2-tailed).

^b^Chi-square test.

### Within-Group Analysis

The within-group analysis presented in Table 2 demonstrates significant improvements in all primary outcomes, namely physical activity, FoC, and QoL among participants in the intervention group over the study period. From baseline (T0) to immediately postintervention (T1), there were moderate to large effect size improvements in physical activity (Cohen *d*=0.65), reduction in FoC (Cohen *d*=0.52), and enhancement in QoL (Cohen *d*=0.60), all statistically significant (*P*<.001). These improvements were not only maintained but further enhanced by the 1-month follow-up (T2), with slightly larger effect sizes observed between T0 and T2 (physical activity: Cohen *d*=0.72; FoC: Cohen *d*=0.56; and QoL: Cohen *d*=0.68). Importantly, the additional comparison between T1 and T2 revealed small but meaningful effect sizes (physical activity: Cohen *d*=0.18; FoC: Cohen *d*=0.21; and QoL: Cohen *d*=0.26), indicating sustained engagement and continued benefit even after the formal intervention ended. In contrast, the control group did not experience any significant change across any outcome over time.

**Table 2:** Within-group analysis: mean scores and SDs across measurement points.

Variable and group		T0^a^, mean (SD)	T1^b^, mean (SD)	T2^c^, mean (SD)	Effect size, Cohen *d*		
					(T0-T1)	(T0-T2)	(T1–T2)
**Physical activity**							
	Intervention	45.23 (8.12)	58.34 (7.89)^d^	60.12 (7.45)^d^	0.65	0.72	0.18
	Control	44.89 (7.98)	46.12 (8.01)	45.78 (7.92)	N/A^e^	N/A	N/A
**Fear of childbirth**							
	Intervention	85.67 (12.34)	72.45 (11.23)^d^	70.12 (10.89)^d^	0.52	0.56	0.21
	Control	84.89 (11.98)	83.45 (12.01)	83.12 (11.78)	N/A	N/A	N/A
**Quality of life**							
	Intervention	65.34 (9.12)	75.23 (8.89)^d^	77.45 (8.56)^d^	0.60	0.68	0.26
	Control	64.89 (8.98)	65.12 (9.01)	65.34 (8.92)	N/A	N/A	N/A

^a^T0: baseline measurement.

^b^T1: postintervention measurement.

^c^T2: 1-month follow-up measurement.

^d^Significant within-group changes (*P*<.001) compared to baseline (T0).

^e^Not applicable.

### Between-Group Analysis

At baseline (T0), there were no significant differences between the intervention and control groups in physical activity, FoC, or QoL (*P*>.05). The intervention group had significantly higher physical activity levels than the control group at both T1 (Cohen *d*=0.58; *P*<.001) and T2 (Cohen *d*=0.61; *P*<.001), lower FoC scores than the control group at T1 (Cohen *d*=0.47; *P*<.001) and T2 (Cohen *d*=0.50; *P*<.001), and higher QoL scores than the control group at T1 (Cohen *d*=0.55; *P*<.001) and T2 (Cohen *d*=0.59; *P*<.001) (Table 3).

**Table 3:** Between-group analysis: mean scores and SDs at each time point.

Variable and time point		Intervention group, mean (SD)	Control group, mean (SD)	*P* value (between-group)	Effect size, Cohen *d*
**Physical activity**					
	T0^a^	45.23 (8.12)	44.89 (7.98)	.75	N/A^b^
	T1^c^	58.34 (7.89)	46.12 (8.01)	<.001^d^	0.58
	T2^e^	60.12 7.45)	45.78 (7.92)	<.001^d^	0.61
**Fear of childbirth**					
	T0	85.67 (12.34)	84.89 (11.98)	.69	N/A
	T1	72.45 (11.23)	83.45 (12.01)	<.001^d^	0.47
	T2	70.12 (10.89)	83.12 (11.78)	<.001^d^	0.50
**Quality of life**					
	T0	65.34 (9.12)	64.89 (8.98)	.71	N/A
	T1	75.23 (8.89)	65.12 (9.01)	<.001^d^	0.55
	T2	77.45 (8.56)	65.34 (8.92)	<.001^d^	0.59

^a^T0: baseline measurement.

^b^Not applicable.

^c^T1: postintervention measurement.

^d^Significant between-group differences (*P*<.001).

^e^T2: 1-month follow-up measurement.

## Discussion

### Principal Findings

The findings indicate that the smartphone app–based exercise management program led to significant improvements in physical activity levels among pregnant women in the intervention group compared to the control group. These positive effects persisted at the 1-month follow-up (T2), suggesting the intervention’s potential for long-term benefits. The growing popularity of smartphone apps for physical activity can be attributed to their user-friendly design and accessibility, which enhance user engagement and adherence to exercise routines [48]. The observed increase in physical activity among the intervention group underscores the effectiveness of mHealth tools in driving behavioral change [49]. This study highlights the promise of a smartphone-based exercise app that combines structured physical activity with behavioral strategies such as MI to encourage exercise among pregnant women [50].

Research indicates that behavioral interventions proven effective outside of pregnancy may also benefit pregnant women. Techniques such as self-monitoring, goal-setting, regular feedback, and MI have demonstrated success in promoting healthier lifestyle behaviors [51,52]. However, there is a lack of research on adapting these interventions for low-income or minority pregnant women, who could benefit from more accessible, home-based, or phone-delivered support [53]. According to the American Congress of Obstetricians and Gynecologists, pregnant women without medical complications should aim for 30 minutes of moderate physical activity most days of the week [1]. One limitation of the app used in this study is the absence of a social-support feature. Incorporating a peer-support network or virtual community could enhance engagement, especially for women facing social or emotional challenges during pregnancy [38]. Another limitation is the relatively short duration of the intervention. While physical activity levels increased in the intervention group over time, a longer follow-up period could reveal whether these improvements are sustained postpartum. Studies suggest that healthy habits developed during pregnancy can persist after childbirth, offering long-term benefits for both maternal and child health [53]. Future research could extend the duration of app use to explore its long-term impact on physical activity behavior. This study adds to the growing evidence supporting the role of digital tools in maternal health, emphasizing the potential of mHealth technology to promote healthy behavior among pregnant women.

The study also found that the smartphone app-based exercise management program significantly reduced FoC among pregnant women in the intervention group compared with the control group. These effects were maintained at the 1-month follow-up (T2), indicating the intervention’s potential for long-term benefits. Previous research has shown that pregnant women who engage in regular exercise experience greater reductions in FoC compared with those who only attend traditional childbirth classes [38,54]. This study demonstrates that a structured physical activity program combined with MI can effectively reduce childbirth anxiety, a critical factor in maternal mental health. Exercise helps alleviate FoC by managing respiratory and muscle tension and reducing overall anxiety [41]. MI, a client-centered approach to addressing ambivalence, further supports physical activity adoption during pregnancy, promoting adherence and positive outcomes [55,56].

Exercise stimulates endorphin release, improves body image, and fosters a sense of control, all of which contribute to reduced FoC [57]. Regular physical activity during pregnancy has been shown to reduce stress and build resilience against anxiety [58]. FoC can lead to adverse outcomes such as higher rates of cesarean sections, postpartum depression, and difficulties with maternal bonding [59]. With the increasing use of smartphones and mHealth, apps provide a flexible and accessible way to deliver interventions, particularly for pregnant women who may face barriers to traditional in-person counseling [60].

The improvements in QoL observed in this study align with previous research showing that structured exercise programs enhance physical and emotional well-being during pregnancy [61]. However, this study extends the evidence by incorporating a culturally adapted, app-based approach, which may be more scalable and accessible in low-resource settings. The integration of MI and interactive features, such as exercise videos and self-assessment tools, likely contributed to the high engagement and adherence rates observed. The inclusion of MI in the app provided pregnant women with the encouragement and support needed to maintain regular exercise, making it a valuable tool for improving QoL [62]. By fostering self-efficacy and motivation, MI can transform childbirth from a fear-inducing event into a manageable and even positive experience [63]. Research suggests that combining physical activity with psychological interventions amplifies their benefits [64]. The app-based approach likely benefited from the synergy between structured exercise and MI, offering a comprehensive strategy for addressing FoC by addressing both physical and mental health needs.

This study examined the effectiveness of a smartphone-based exercise management app integrated with behavioral intervention techniques, particularly MI, on improving QoL scores. Systematic reviews have shown mixed but promising results regarding exercise interventions for pregnant women. Previous research indicated that combined exercise programs positively influenced QoL in 2 of 3 studies, and all studies involving yoga or physical activity suggested potential QoL benefits for pregnant women [65]. Despite some inconsistencies, this body of evidence supports the idea that well-structured exercise programs can effectively enhance QoL during pregnancy.

The observed improvements in QoL reflect the growing recognition of digital-health interventions as tools for improving health outcomes. In recent years, digital-health technologies, such as smartphone apps, have gained attention for their ability to provide convenient, personalized, and accessible support for managing health-related behaviors [66]. These technologies enable individuals to engage in health-promoting behaviors more effectively by offering continuous feedback and motivation, which are essential for sustaining behavior change. For pregnant women facing physical and emotional challenges, an exercise-management app that incorporates MI can serve as a powerful tool for promoting a healthier lifestyle and improving mental well-being [65]. This study highlights the potential of a smartphone-based exercise-management app, combined with MI, as a valuable resource for enhancing QoL among pregnant women. The integration of structured exercise routines with behavior-change techniques provides a holistic approach to addressing the physical and psychological demands of pregnancy [64]. While further research is needed to optimize these interventions and assess their long-term effects, the current findings suggest that digital-health tools can play a significant role in supporting maternal health. By empowering pregnant women to take an active role in their well-being, mHealth apps represent a promising advancement in prenatal care.

### Implications for Clinical Practice and Public Health

First, the smartphone app–based intervention offers a scalable and cost-effective solution to address the growing burden of maternal health challenges in resource-limited settings. By leveraging widely available mobile technology, this approach can overcome barriers such as limited access to health care facilities and trained professionals. Second, the intervention’s focus on physical activity, FoC, and QoL addresses multiple interconnected dimensions of maternal well-being. For example, increased physical activity may indirectly reduce FoC by improving overall health and self-efficacy, while enhanced QoL may encourage adherence to exercise routines.

Health care providers can integrate such interventions into routine prenatal care, particularly for women at risk of sedentary lifestyles, high FoC, or poor QoL. The app’s modular design, which includes educational content, exercise videos, and MI, can be customized to meet the specific needs of diverse populations. Additionally, the app’s backend data-collection capabilities enable real-time monitoring of participant engagement and outcomes, facilitating timely interventions and personalized feedback.

### Limitations

Despite its strengths, this study has several limitations. First, the quasi-experimental design limits the ability to establish causality, as unmeasured confounding factors may have influenced the results. Future studies should use randomized controlled trials (RCTs) to strengthen the evidence base. Second, the sample was recruited from a single region in Indonesia, which may limit the generalizability of the findings to other populations or cultural contexts. Third, the reliance on self-reported measures, such as the PPAQ and WDEQ-A, may introduce response bias.

Future studies should consider extending the follow-up period beyond 1 month to assess the long-term sustainability of improvements in physical activity, FoC, and QoL. Understanding whether behavioral and psychological gains persist into the third trimester, delivery, and postpartum period would offer valuable insights into the intervention’s lasting impact. Additionally, while this study demonstrated effectiveness among low-risk pregnant women, future research should evaluate the intervention’s safety, usability, and outcomes among women with high-risk pregnancies, including those with gestational complications or comorbidities. Moreover, qualitative studies are encouraged to explore participants’ lived experiences, preferences, and perceived barriers to engagement with the app, which could inform more personalized and culturally nuanced intervention designs. Further development of the digital platform could also include real-time clinical feedback, integration with wearable devices for physical activity tracking, and multilingual support to improve accessibility in other LMIC contexts. Finally, large-scale RCTs with diverse geographic and sociodemographic populations are warranted to validate the generalizability of the findings. Collaborations with policymakers and public health authorities will be essential to evaluate cost-effectiveness and guide potential integration of such digital interventions into national antenatal care programs.

### Conclusions

This study provides robust evidence supporting the effectiveness of a smartphone app–based exercise management intervention in improving physical activity, reducing FoC, and enhancing QoL among pregnant women in Indonesia. The intervention’s scalability, accessibility, and sustained benefits make it a promising tool for addressing maternal health challenges in resource-limited settings. Future research should focus on RCTs, longer follow-up periods, and diverse populations to further validate these findings and explore the intervention’s broader applicability. By integrating mHealth solutions into prenatal care, health care systems can improve maternal health outcomes and reduce disparities in access to quality care.

## Abbreviations

API: application programming interface
CBT: cognitive-behavioral therapy
CFA: confirmatory factor analysis
CFI: comparative fit index
CONSORT: Consolidated Standards of Reporting Trials
FoC: fear of childbirth
ICC: intraclass correlation coefficient
LMICs: low- and middle-income countries
mHealth: mobile health
MI: motivational interviewing
PHC: public health center
PPAQ: Pregnancy Physical Activity Questionnaire
QoL: quality of life
QOL-GRAV: Quality-of-Life Gravidarum
RCT: randomized controlled trial
RM-ANOVA: repeated-measures analysis of variance
RMSEA: root-mean-square error of approximation
SDK: software development kit
STIKep PPNI: Sekolah Tinggi Ilmu Keperawatan Persatuan Perawat Nasional Indonesia
T0: baseline measurement
T1: postintervention measurement
T2: 1-month follow-up measurement
WDEQ-A: Wijma Delivery Expectation Questionnaire Version A

## Data Availability

The datasets generated and/or analyzed during this study are not publicly available due to participant confidentiality and institutional data protection policies but are available from the corresponding author upon reasonable request.
